# International comparison of neutrophil surface markers after severe trauma is feasible using point of care flowcytometry - results from a pilot study

**DOI:** 10.1007/s00011-026-02335-1

**Published:** 2026-07-31

**Authors:** Christian Thomas Hübner, Emma de Fraiture, Nathalie Piaz, Louise Virginie Duebel, Roman Pfeifer, Sanne Brands, Felix Karl-Ludwig Klingebiel, Yannik Kalbas, Paolo Cinelli, Leo Koenderman, Falco Hietbrink, Hans-Christoph Pape, Michel Paul Johan Teuben

**Affiliations:** 1 Department of Traumatology, University Hospital Zurich, University of Zurich, Raemistrasse 100, Zurich, 8091 Switzerland; 2 Department of Trauma Surgery, University Medical Center Utrecht, Utrecht, Netherlands; 3 Center for Translational Immunology (CTI), University Medical Center Utrecht, Utrecht, Netherlands; 4 Department of Respiratory Medicine, University Medical Center Utrecht, Utrecht, The Netherlands

**Keywords:** Polytrauma, Neutrophils, Point-of-care flow cytometry, Immune monitoring, CD16/CD11b/CD10, Multicenter validation

## Abstract

**Introduction:**

The dynamics of cell surface marker expression on systemic neutrophils provide valuable insights into the post-traumatic immune response. Recent advances in point-of-care (PoC) technologies allow for rapid on-site neutrophil analysis. The current study aimed to proof, if results of PoC measurements are comparable between two study centers.

**Methods:**

A well-established laboratory protocol for fully automated PoC flowcytometry analysis of neutrophils was applied inside the trauma bay of two level 1 trauma centers. We analyzed the neutrophil surface patterns CD16, CD11b, CD62L, CD10 and CD64 and compared the results between two centers both in heathy controls and patients.

**Results:**

28 Polytrauma patients were included. The measurements at both centers revealed similar results with respect to the expression neutrophil activation markers in patients with different trauma severities. Particularly the expression of CD16, CD11b and CD10 were very comparable between both centers in both healthy controls and trauma patients.

**Conclusion:**

This study demonstrates the successful implementation of a fully automated PoC neutrophil analysis infrastructure from one trauma center to another facilitating multicenter flow cytometry studies. This paves the way for advanced immune monitoring that could significantly improve personalized clinical decision-making.

## Introduction

Trauma represents a main cause for morbidity and mortality in the young [1]. Since improved resuscitation strategies and surgical techniques have led to a decreased mortality due to exsanguination, the relative importance of inflammatory and infectious complications has increased as more patients survive the initial phase of trauma [2]. Neutrophils are believed to play a crucial role in the development of inflammatory complications, such as multiple organ dysfunction (MODS), acute respiratory distress syndrome (ARDS) and sepsis [3–5]. Several markers on neutrophils have been linked with impaired outcome after trauma. Especially the parameters FcgRIII/CD16, Mac-1/CD11b, L-Selectin/CD62L, neutral endopeptidase/CD10 and FcgRI/CD64 have come into focus in recent studies [6–8]. Novel advances in fully automated point-of-care (PoC) technologies allow for rapid near patient flow analyses including PoC studies on neutrophil characterization. A standardized protocol for fully automated PoC analysis of neutrophils has been established at the trauma bay and validated in a trauma center in the Netherlands [7, 9].

This study aimed to validate the standardized protocol at another international centerand assess the feasibility of transferring both the protocol and associated logistics from The Netherlands to a second center in Switzerland.

### Methods

### Study design and population

This study has been performed at two European level one trauma centers in Zurich (Switzerland) and Utrecht (the Netherlands). In both study centers ethical approval (Zurich: BASEC 2017 − 01380, Utrecht: PREDICT 22U-0385 and AQUIval 25U-0305), as well as patient informed consent were obtained. A group of eight healthy volunteers in Zurich was sampled in parallel with the severely injured trauma patients. In Utrecht blood samples from eight healthy volunteers were obtained from the institute Mini Donor Service (Protocol-No 07-125/C).

### Automated flow cytometry

Blood samples were collected and immediately (< 0.5 h) analyzed using the fully automated AQUIOS CL “load & go” flow cytometer (Beckman Coulter Life Sciences, Miami, FL, USA). The flow cytometer in Zurich was installed and calibrated in accordance with the standardized protocol settings from Utrecht [7, 9]. Needle positioning, laser settings, and compensation parameters in Zurich were adapted to predefined values from Utrecht. The device automatically pipettes blood from a tube into a 96-well plate, followed by staining with an 18 µL customized antibody panel containing CD16-FITC (clone 3G8), CD11b-PE (clone Bear1), CD62L-ECD (clone DREG56), CD10-PC5 (clone ALB1), and CD64-PC7 (clone 22). After 15 min, red blood cells were lysed with 335 µL AQUIOS Lysing Reagent A and stopped with 100 µL AQUIOS Lysing Reagent B, before analysis in the flow cell. The results were exported as FCS 3.1 High Res Listmode Files (.lmd) and imported to FlowJo (De Novo Software, Glendale, CA, USA, Version 10.10.0). Granulocytes were manually gated based on forward and sideward scatter. Statistical analysis was performed using R-Studio (R Foundation for Statistical Computing, Vienna, Austria, Version 2024.12.1 + 563). A two-sided Mann-Whitney-tests with a significance level of 0.05 was used. Diagrams were designed using GraphPad Prism (GraphPad Software, Boston, United States, Version10).

## Results

In this study, 28 polytrauma patients with a median ISS of 29 (IQR 26.75-38) were included. Fourteen patients from Utrecht and Zurich each were compared. The demographic parameters of the patients are shown in Table 1.

**Table 1:** Patient demographics and -characteristics

	All patients	Zurich	Utrecht	Statistics
Total	28	14	14	
Sex				
Male	17	9	8	
Female	11	5	6	
Age				
Mean (SD)	55.1 (± 18.6)	62.9 (± 19.3)	47.4 (± 14.8)	*p* = 0.02
Median (Range)	57.5 (19–83)	64.5 (19–83)	48.5 (22–67)	*p* = 0.03
IQR	44.75–66.25	56.25-79	40.5-60.25	
ISS				
Mean (SD)	32.5 (± 7.8)	32.4 (± 8.9)	32.6 (± 6.7)	*p* = 0.96
Median (Range)	29 (25–51)	29 (25–51)	29.5 (26–48)	*p* = 0.46
IQR	26.75-38	26-38.25	29–37	

Measurement of neutrophil surface expression patterns were conducted as soon as possible after appearance in trauma bay. In Zurich median time between accident and measurement (including transport to the hospital) was 177 min, whereas in Utrecht the median time was 91 min. Time between arrival in trauma bay and measurement was in median 68 min in Zurich and 29 min in Utrecht, respectively.

### Comparison MFI CD10, CD16 and CD11b between trauma and control

**Fig. 1:**
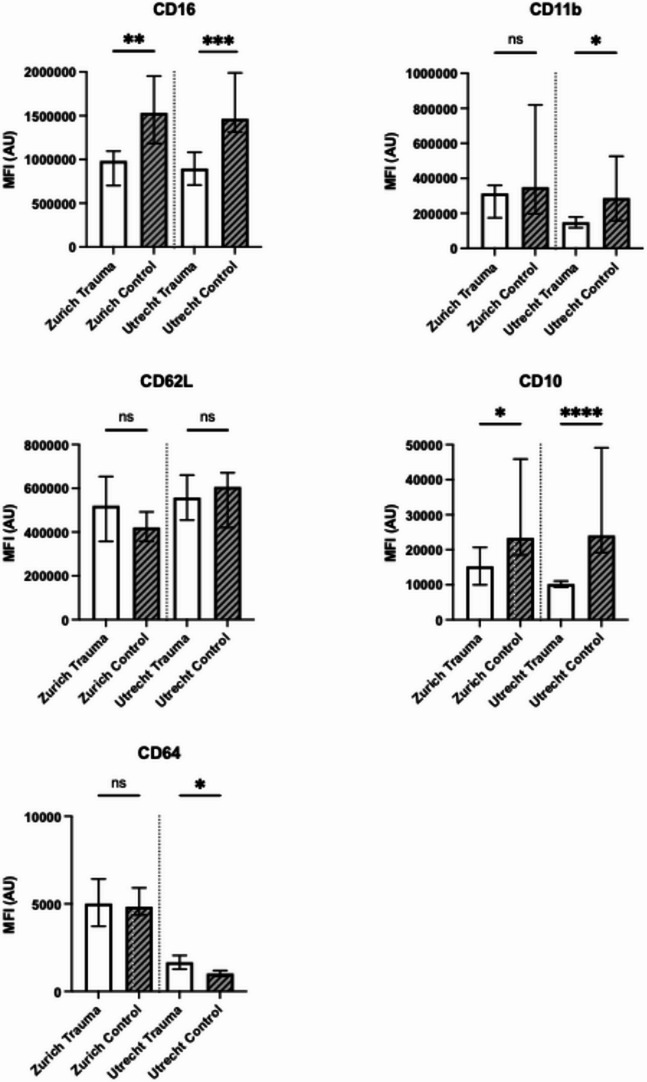
MFI of neutrophil surface markers, comparing Zurich and Utrecht in trauma and control groups. White bars indicate trauma patients; grey bars indicate healthy controls

In both groups significant differences in expression of CD10, CD11b and CD16 were observed between trauma and control. For CD10 the median expression in the trauma group was 35% and 59% lower (*p* = 0.024, *p* < 0.0001) compared to healthy controls in Zurich and Utrecht, respectively (Fig. 1). For CD16 the difference was 36% and 43% (*p* = 0.003 and *p* = 0.0003) and for CD11b 10% and 40% (*p* = 0.212, *p* = 0.013). There was no statistical significance in expression of CD11b on neutrophils between patients and controls in the Zurich group.

### Comparison expression between both centers

When comparing median expression values for CD10, CD11b and CD16 between Zurich and Utrecht, no significant differences appeared (*p* = 0.87, *p* = 0.54 and *p* = 0.95). On the other hand, for CD62L and CD64 the median MFI was significantly different between both cities (*p* = 0.02 and *p* = 0.004, respectively).

## Discussion

Neutrophil receptor/marker expression patterns give valuable insights into alterations of the immune system after trauma. While measurement of neutrophil expression patterns historically needed time and were cost intensive measurements, recent advances in PoC diagnostic flow methods allow for a rapid and standardized use in the trauma bay. However, flow cytometry outcomes are known for being hard to compare between different devices as MFI depends on factors such as laser power, detector sensitivity, filter configurations, and compensation settings [10].

In this validation study, we demonstrated that a fully automated flow cytometer could be set up and calibrated in a second center. Due to the standardized set up and the alignment of the lasers with beads, it was possible to compare and pool data from two trauma centers. We found no statistical difference in the MFI values of the relevant expression patterns of CD16, CD11b CD10. However, for CD62L and CD64 there were statistically significant differences. Next to small but relevant differences in the technical set-up of the flow cytometers, the observed differences could be due to ex vivo stressors (e.g. venipuncture procedures), minor differences in sample handling, or true biological variability between patient populations.

Between patients and control group significant differences for the markers of CD16, CD11b and CD10 were detected [8]. CD16 is known as a maturity indicator and reflect the release from young cells from the bone marrow to the blood [8, 11]. CD11b is both an activation and maturation marker as CD11b is expressed less on immature granulocytes normally found in the bone marrow, which can be released due to severe multitrauma [12]. On the other hand, CD11b is an activation marker that is highly expressed due to trauma [7, 13]. This double function might explain the wide range in expression in the Zurich cohort, as well its insignificance compared to the control group.

## Conclusion

In conclusion, the current study demonstrates that PoC neutrophil analysis in the trauma bay is feasible in a multicenter approach to monitor the early post-traumatic immune response. The study validates the standardized sampling method and shows that pooling and comparison of data from different international institutes is feasible.

This opens up the possibility for multicenter studies to combine flow cytometry data from different study centers.

## Data Availability

The generated dated of this study are available from the corresponding author on reasonable request.
